# Longitudinal Examination of Loneliness and Smoking in Adolescents and Young Adults: A Multiple Dataset Study

**DOI:** 10.1007/s11469-025-01522-1

**Published:** 2025-08-05

**Authors:** Maaike Verhagen, Desi Beckers, Nina van den Broek, Kirsten J. M. van Hooijdonk, Suhaavi Kochhar, Laila Qodariah, Milagros Rubio, Eveline Sarintohe, Jacqueline M. Vink

**Affiliations:** Behavioural Science Institute, Radboud University, Thomas van Aquinostraat 4, Nijmegen, 6525 GD Netherlands

**Keywords:** Smoking, Loneliness, Adolescents, Young adults, Multi-dataset

## Abstract

Loneliness might be associated with smoking, especially in youth. We examined temporal associations from loneliness to smoking behaviours among adolescents and young adults using a multi-dataset approach. For adolescents (sample 1: *N* = 600, *M*_age_ = 12.6, 52.7% female; sample 2: *N* = 551, *M*_age_ = 12.8, 54.3% female; sample 3: *N* = 261, *M*_age_ = 13.8, 46.7% female), discrete-time survival analyses revealed that loneliness did not predict smoking initiation over time. For young adults (sample 4: *N* = 2,636, *M*_age_ = 21.7, 70.5% female; sample 5: *N* = 404, *M*_age_ = 22.6, 65.6% female), structural equation models revealed that baseline loneliness did not predict smoking status (non-smoker/occasional smoker/regular smoker) after 6 and 18 months (except in sample 4 after 18 months; *β* = 0.07, *p* = 0.035). Complementary analyses showed that baseline loneliness preceded regular but not occasional smoking. Utilizing multiple datasets enabled focusing on overall patterns and obtaining robust insights. Future studies could explore at-risk subgroups. With complex behaviours like smoking, many risk factors play a role, each having small effects.

**Supplementary Information:**

The online version contains supplementary material available at 10.1007/s11469-025-01522-1.

Adolescence and young adulthood are periods of major development that shape future patterns in adult mental and physical health (National Academies of Sciences, Engineering, and Medicine, 2019; Sawyer et al., 2012). During these years, individuals undergo significant biological, psychological, and social developments (like brain maturations and changes in social context) (Andrews et al., 2020; Smetana et al., 2006). Given these developments, individuals who transition from adolescence to young adulthood are specifically vulnerable to experiencing feelings of loneliness (Qualter et al., 2010, 2015). Loneliness is defined as a mismatch between desired and actual social relationships, in either quality, quantity, or both (Perlman & Peplau, 1981), which arises when individuals perceive their social relationships as insufficient in meeting their social needs (Hawkley & Cacioppo, 2010). Several factors have been identified as predictors of loneliness. These include adverse social experiences such as bullying and poor family relationships, as well as lower levels of social support. Moreover, individual characteristics such as shyness, depression, and low self-esteem have also been linked to increased loneliness (Bayat et al., 2021; Buecker et al., 2024; Mahon et al., 2006).

Loneliness is highly prevalent among young people (RIVM, 2023; Trimbos-instituut, 2024) and is increasingly recognized as a public health concern (Holt-Lunstad et al., 2015). In the Netherlands, ~ 12% of adolescents usually or always feel lonely (HBSC, 2023). Simultaneously, loneliness among young adults, e.g. university students, is also of increasing concern (Ellard et al., 2023; Werner et al., 2021). A noteworthy number of university students in the Netherlands report severe loneliness, ranging from 23% (Van der Heijde et al., 2018) to 30% (Caring Universities Consortium, 2023).

The transitions that individuals encounter during adolescence and young adulthood might trigger adolescents to start engaging in risk-taking behaviours such as tobacco smoking (Castro et al., 2023). Research showed that in 2023, 16% of 12–16-year-olds in the Netherlands had ever smoked, and 8.5% had smoked in the last month (Trimbos-instituut, 2024). Prevalences were stable in the past years. In young adults, the number of smokers decreased in the past years, and in 2023, the percentages of smokers were 18.8%, 28.1%, and 29.3% in 18–19-year-olds, 20–24-year olds, and 25–29-year-olds, respectively (Trimbos-instituut, 2024).

## Cross-Sectional Studies on Loneliness and Smoking Behaviours

Both loneliness and tobacco smoking can significantly impact development during the vulnerable period of adolescence and young adulthood and set the stage for lifelong health trajectories. Previous cross-sectional studies have explored their co-occurrence and show mixed findings. Regarding adolescents, a systematic review by Dyal and Valente (2015) reported a positive association between being a current smoker and higher levels of loneliness in 6 out of 11 adolescent samples. This positive association was also reported in more recent cross-sectional studies among adolescents (McClure-Thomas et al., 2022; Shadmehr et al., 2019). Regarding young adults, several studies provided some support for a positive association between loneliness and smoking among university students or reported lonely students to report more tobacco use (Habibi et al., 2018; Peltzer & Pengpid, 2017; Zahedi et al., 2022, 2024; Zhang et al., 2020), while other studies did show that smoking (vs. non-smoking) university students did not differ on loneliness (Anjum & Smitha, 2020; Diehl et al., 2018). Consequently, previous studies on cross-sectional associations between loneliness and smoking among adolescents and young adults presented conflicting insights.

## Longitudinal Studies on Loneliness and Smoking Behaviours

Cross-sectional studies cannot disentangle the temporal relationship between loneliness and smoking. In this study, we focused on the longitudinal relationship from loneliness to smoking behaviour among adolescents and young adults. So far, only a few longitudinal studies have assessed this longitudinal relationship in these populations. According to Park (2009), loneliness in school predicting smoking initiation among Korean elementary-school students (4th grade onwards) in bivariate analyses, while the findings were not robust in multivariate analysis controlling for other factors. Qualter et al. (2013), examined different trajectories of loneliness (age 7 to 17) but did not find support for any trajectory predicting smoking status (yes/no). Although these studies provide valuable insights into possible longitudinal associations between loneliness and smoking initiation/status among adolescents/young adults, methodological limitations (e.g. self-reporting, significant drop-outs, bias by uncontrolled confounding) might have impacted the findings. Consequently, more longitudinal studies, across different cultures, are needed to confirm the findings from these two existing longitudinal studies.

Other available studies focused on older samples, e.g. working adults (Stenlund et al., 2021) or older adults (> 50 years; Luo & Waite, 2014; Philip et al., 2022), or utilized other study designs. For instance, a Mendelian randomization study, where genetic variants are utilized as instrumental variables to assess potential causal effects (Richmond & Davey Smith, 2022), did report weak evidence for increased loneliness leading to higher likelihood of initiating smoking, smoking more cigarettes, and a lower likelihood of quitting smoking in adults (Wootton et al., 2021).

Taking this together, limited research showed no or weak evidence for a longitudinal relationship from loneliness to smoking (in adolescents or adults), and as far as we know, no study has specifically focused on young adults (e.g. university students). Additionally, it is important to disentangle how loneliness might be longitudinally associated with different smoking behaviours, i.e. smoking initiation, being a current smoker (smoking status), or smoking frequency (among smokers). Consequently, additional longitudinal studies are needed to shed new light on the possible longitudinal relationship from loneliness to smoking among adolescents and young adults, considering different smoking behaviours. These insights are essential, as longitudinal studies can yield new insights that can facilitate the development of interventions that address loneliness and reduce smoking behaviour among youth (Stickley et al., 2014).

## Potential Underlying Mechanisms for the Positive Relation Between Loneliness and Smoking

Although the literature on possible cross-sectional and longitudinal associations between loneliness and smoking is mixed, several mechanisms have been proposed explaining possible positive associations among adolescents/young adults. On the one hand, isolated individuals (compared to cliques or liaisons) might be more likely to smoke based on motivational processes aimed at enhancing belongingness and connection (Seo & Huang, 2012). People who experience feelings of loneliness may smoke or initiate smoking to increase opportunities for social contact (DeWall & Pond Jr., 2011). Possibly, people might try to reconnect through mimicking behaviours such as smoking to gain social acceptance. These ideas are based on evidence that people who feel lonely show lower inhibition to perform behaviours detrimental to health, such as smoking (DeWall & Pond Jr., 2011).

On the other hand, coping mechanisms, such as the self-medication hypothesis, might explain why individuals who are experiencing loneliness might use cigarettes as a remedy for distress or alleviate the painful emotions associated with loneliness (Brennan, 1982; Khantzian, 1985; Mischel et al., 2014; Munafò et al., 2008).

## Current Study

In the current study, we examined longitudinal associations between loneliness and smoking behaviour. We adopted a multi-dataset approach, exploring these longitudinal associations in multiple samples of adolescents and young adults. This is a unique approach, which on the one hand allows us to compare findings across different samples of the same developmental stage, providing a more robust answer, which is highly valuable given the mixed results in the literature. On the other hand, it allows us to compare different developmental groups and investigate development-specific factors that contribute to smoking. This is highly valuable, as these can be used to tailor prevention and intervention services to the needs of specific developmental groups.

The aim of this study was twofold. First, using three adolescent samples (12–18 years), we examined whether adolescent loneliness is longitudinally associated with smoking initiation. We chose to focus on smoking initiation in this developmental period since this is the stage at which smoking behaviour is usually initiated. We hypothesized that adolescents who feel lonely will be more likely to start smoking than adolescents who feel less lonely. Second, in two university student samples (18–30 years), we investigated if loneliness predicted smoking status (i.e. non-smoker, occasional smoker, regular smoker) at 6 and 18 months after baseline. We chose to examine smoking status—and not initiation—since we expect that by this age, fewer novice smokers are to be expected and established smoking status is more relevant among this older age group. We expected that participants who feel lonely at baseline would be more likely to have an established status at the next time point(s) compared to participants who feel less lonely at baseline.

## Methods

### Pre-Registration

The research questions, hypotheses, and planned analyses were pre-registered prior to data analyses: https://osf.io/946xu (adolescent samples) and https://osf.io/p79dm (student samples). Deviations are reported in Supplementary Material I.

### Participants

This study was conducted following the principles of the Declaration of Helsinki. For all samples, participants provided informed consent before inclusion, and ethical approval was obtained. A short description of the included samples can be found below (for more information, see Supplementary Material II).

#### Sample 1: Kandinsky Longitudinal Study (KLS)

KLS is an ongoing project on Dutch adolescents’ socio-emotional well-being with annual waves of data collection of students in grades 7–10 (https://osf.io/8ztk5/). For this study, we included 828 adolescents from three cohorts: 7th-grade students in 2012 (cohort 1, *n* = 284), 2013 (cohort 2, *n* = 259), and 2014 (cohort 3, *n* = 286). At baseline, all students were in 7th grade (first year of secondary school in the Dutch educational system). Data from 196 adolescents were removed because they initiated smoking before baseline (*n* = 34), had missing data at baseline (*n* = 162), or did not have smoking information at follow-up (*n* = 32). Thus, the analytic sample consisted of 600 adolescents (cohort 1: *n* = 242, cohort 2: *n* = 177, cohort 3: *n* = 181). At W1, the sample included 52.7% females and 47.3% males. The mean age at W1 was 12.59 years.

#### Sample 2: G(F)OOD Together project (GFT)

GFT is a 6-wave longitudinal study on Dutch adolescents’ and their parents’ health behaviours (van den Broek et al., 2020, 2022); https://osf.io/bysgq/). At baseline (W1), students were in 7th or 8th grade. W1 and 3 took place before the COVID-19 pandemic (fall 2017, *n* = 667; spring 2019, *n* = 674), and W4 to W6 during the COVID-19 pandemic (i.e. fall 2020, *n* = 306; spring 2021, *n* = 142; fall 2021, *n* = 129). As loneliness with peers was not assessed in W2, this wave (and corresponding data) will not be used in the current study. At W4, adolescents were asked to retrospectively report about the first COVID-19 lockdown (W4 retro, spring 2020, *n* = 306). Data from 232 adolescents were removed because they initiated smoking before W1 (*n* = 28), had missing data at baseline (*n* = 131), or did not have smoking information at follow-up (*n* = 73). The analytic sample consisted of 551 adolescents. At W1, the sample included 54.3% females and 45.7% males. The mean age at W1 was 12.81 years.

#### Sample 3: Family & Health study (F&H)

F&H investigated socialization processes underlying health behaviours (Harakeh et al., 2005). In this study, 428 Dutch families were included with at least two children aged 13–16 years. Six waves of data were collected over 5 years. Baseline (W1) was collected between November 2002 and April 2003. The number of families at the yearly follow-up waves was 416 (W2), 404 (W3), 356 (W4), 326 (W5), and 313 (W6). In this project, data from the youngest sibling was selected. Data from 167 adolescents were removed because they initiated smoking before W1 (*n* = 153), had missing data at baseline (*n* = 8), or did not have smoking information at follow-up (*n* = 6). The analytic sample consists of 261 adolescents. At W1, the sample included 46.7% females and 53.3% males. The mean age at W1 was 13.80 years.

#### Sample 4: Healthy Student Life project (HSL)

HSL (van Hooijdonk et al., 2023; https://osf.io/e4d5b/) is a longitudinal online survey study that investigates the well-being, lifestyle, and individual and contextual factors of Dutch university students. So far, three waves of survey data have been collected (W1 fall 2021, *n* = 4.902; W2 spring 2022, *n* = 4.431; W3 spring 2023, *n* = 5.214). At each wave, all students enrolled at Radboud University (± 25.000) were invited to participate. In addition, at W3, students who graduated after W2 and gave permission for a follow-up were invited (W3 alumni, *n*_invited_ = 484). After the exclusion of participants above 30 years old, with no longitudinal data (participation at W1 and at least W2 or W3 was required) or who reported inconsistently on smoking, the analytic sample consisted of *n* = 2.636 (W1), *n* = 2.168 (W2), and *n* = 1.666 (W3) participants, with complete data on both smoking and loneliness. At W1, the sample included 75.2% females, 23.3% males, and 1.5% gender other. The mean age at W1 was 22 years.

#### Sample 5: al-RISCO lifestyle project (al-RISCO)

Al-RISCO (Rubio et al., 2024; https://osf.io/urhdt/) is a two-year longitudinal survey study (early 2021–May 2023) to examine the cognitive, social, and emotional factors of Dutch university students’ lifestyles who reported heavy (episodic) drinking before the COVID-19 outbreak. Out of 1176 students screened (fall 2020), 634 students were classified as heavy drinkers and were invited to participate. The sample includes students of applied and research universities who were in the last year of their bachelor studies or any year of their master studies at the time of screening. For the current study, three waves of data were selected: W1 (spring 2021, *n* = 439), W2 (fall 2021, *n* = 415), and W3 (fall 2022, *n* = 366). After the exclusion of participants who reported inconsistently on smoking, the analytic sample consisted of *n* = 404 (W1), *n* = 377 (W2), and *n* = 326 (W3) participants, with complete data on both smoking and loneliness. At W1, the sample included 65.6% females, 33.9% males, and 0.5% gender other. The mean age at W1 was 22.55 years.

## Materials

All measures and coding are described in Supplementary Material III. A brief explanation is provided below.

### Loneliness

In the adolescent samples, the Loneliness and Aloneness Scale for Children and Adolescents (LACA) subscale ‘loneliness’ in relation to peers was used (Goossens & Maes, 2020). A mean score was calculated for the 12 items (range 0–3). Cronbach’s alpha for W1–5 varied between 0.86 and 0.94. In HSL, the Roberts UCLA Loneliness Scale (RULS-8) was used (Goossens et al., 2014). A mean score of the eight items was calculated (range 0–4) for all participants who answered at least 80% of the items. Cronbach’s alpha for W1 was 0.87. In al-RISCO, the Three-Item Loneliness Scale (UCLA-revised: (Hughes et al., 2021; Russell et al., 1980)) was used. A mean score of the three items (range 1–3) was calculated for participants who completed the whole scale. Cronbach’s alpha for W1 was 0.69. Higher scores in all samples indicated more loneliness.

### Smoking

In the adolescent samples, various questions on smoking initiation or smoking status were used to categorize participants into smoking initiation no (coded as 0) or yes (coded as 1) (see Supplementary Material III). For each wave after baseline, if participants indicated they never smoked, smoking initiation was coded as 0, while if participants indicated they had ever smoked, smoking initiation was coded as 1 (and the following waves of data were removed from the dataset). We chose to operationalize smoking as smoking initiation in the adolescent samples since adolescence is a period where most adolescents have not yet progressed to regular smoking, and therefore, measuring initiation allows us to capture the onset of smoking behaviour. For the university student samples, a question on smoking frequency was used to define participants’ current smoking status as ‘non-smoker’ (coded as 0; smoking frequency was never, a few puffs, or former), occasional smoker (coded as 1; smoking frequency was monthly or less), and regular smoker (coded as 2; smoking frequency was weekly/daily). We chose to operationalize smoking as smoking status since young adults in university have likely either initiated smoking or had the chance to experiment with it. Smoking status would allow us to understand the amount of smoking beyond a binary value of yes or no.

### Other Variables

In the adolescent samples, biological sex (boy or girl) was asked, while in the university student samples, gender (male, female, other) was asked. In all samples, age at W1 was either asked in the questionnaire or calculated based on the date of participation and date of birth. For samples 2 and 3, educational type was categorized into three levels: (1) pre-vocational education, (2) higher general secondary education, and (3) pre-university education. For sample 5 (al-RISCO), the educational type was categorized into two levels: (1) applied and (2) research university.

## Analyses

All analyses were performed in R (R Core Team, 2023; R codes are available via OSF: https://osf.io/4sk5h/). For all samples, descriptive statistics of all study measures were explored.

### Adolescent Samples (Samples 1, 2, and 3)

To examine whether loneliness predicts smoking initiation, we conducted discrete-time survival analyses using logistic regression models. The datasets were transformed into person-period files, where each participant had a separate row representing a time period. Once a participant initiated smoking, the following time points of data for that participant were removed (i.e. censoring). The models were performed with the glm function in R, using the complementary log–log link function. These models contained smoking initiation as the dependent variable, and the following independent variables: time (categorical; 5 time points), loneliness (at t-1; continuous, time-varying), sex (categorical, time-invariant), educational level (categorical, time-invariant, not available in sample 1 (KLS)), and age at baseline (continuous; time-invariant). We used sum-to-zero contrasts for categorical variables (i.e. sex and educational level), with − 1 reflecting boys and the lowest category of educational level. The threshold for statistical significance was set to *α* = 0.05.

### University Student Samples (Samples 4 and 5)

Structural equation modelling (SEM) was used to explore the longitudinal association between baseline (T1) loneliness and smoking status (non-smokers, occasional smokers, or regular smokers) after 6 months (T2—short term) and 18 months (T3—long term). The R package ‘lavaan’ was used (Rosseel, 2011). In the model, loneliness at T1 was added as the independent variable, smoking status at T2 and T3 were added as (ordered) dependent variables, and smoking status at T1, gender, age at T1, and educational level (al-RISCO only) were added as covariates. For HSL, dummy coding was used for gender, while for al-RISCO, the category gender other was disregarded due to small numbers. Before conducting the analyses, missing data were imputed using KNN imputation (Memon et al., 2023). In the model, the weighted least squares mean and variance adjusted (WLSMV) estimator was used to estimate the model parameters (Suh, 2015) and the dependent variables were defined as ordered (lavaan project, 2023; Rosseel, 2011). The threshold for statistical significance was set to α = 0.05. In both samples, sensitivity analyses (with complete cases/smoking quantity (only for the HSL sample)) and attrition analyses were performed (see Supplementary Materials IV and V).

Exploratory analyses (deviation from pre-registration): In the main analyses, smoking status (non-smoker, occasional smoker, regular smoker) was included as an ordered variable (resulting in one beta). To better understand possible contrasts between the different smoking status groups, we have complemented the main analyses in both student samples with two nominal regression analyses, one with the dependent variable smoking status at T2 and one with the dependent variable smoking status at T3. The independent variables were the same as in the main analyses (gender, educational level (al-RISCO only), smoking status at T1, and loneliness at T). With these exploratory analyses, two different betas for occasional and regular smokers (compared to the reference group of non-smokers) were estimated.

## Results

### Adolescent Samples

#### Descriptives

The descriptive statistics of the adolescent samples (i.e. KLS, GFT, and F&H) are described in Table 1. Sex distribution was relatively balanced across the samples: Females constituted 52.7% of the KLS sample, 54.3% of GFT, and 46.7% of F&H. The mean age at T1 was between 12.6 and 13.8 years across samples. The KLS sample consisted of only students from higher general secondary/pre-university education. The GFT and F&H samples also consisted mostly of adolescents in higher educational levels. The mean loneliness is relatively low, as scores vary between 0.35 and 0.56 across all samples on a 0- to 3-point scale. Regarding smoking, it should be noted that 4.1%, 4.2%, and 35.7% smoked at the baseline of the KLS, GFT, and F&H study, respectively, and were excluded from the analyses.

**Table 1:** Descriptive statistics (% or M(SD)) for the adolescent samples

	KLS (*N* = 600)	GFT (*N* = 551)	F&H (*N* = 261)
Gender			
Male	47.3%	45.7%	53.3%
Female	52.7%	54.3%	46.7%
Age T1	12.59 (0.40)	12.81 (0.61)	13.80 (0.39)
Educational type T1			
Pre-vocational	n.a.	32.5%	26.4%
Higher general secondary	n.a.	9.2%	41.3%
Pre-university	n.a.	58.3%	29.9%
Loneliness			
T1	0.47 (0.49)	0.38 (0.45)	0.53 (0.53
T2	0.43 (0.50)	0.35 (0.50)	0.54 (0.53)
T3	0.38 (0.45)	0.52 (0.54)	0.55 (0.57)
T4	0.44 (0.52)	0.45 (0.50)	0.56 (0.58)
T5	n.a.	0.55 (0.48)	0.51 (0.58)

*n.a.* not applicable, *KLS = Kandinsky Longitudinal Study*, *GFT* = G(F)OOD together, *F&H* = Family & Health study

#### Discrete-Time Survival Analyses: How Is Loneliness Longitudinally Associated with Smoking Initiation?

The life table of smoking for the three adolescent samples is shown in Supplementary Material VI. As the hazard ratios were not significant in any of the three adolescent samples, they were not further interpreted (the exact values can be found in this section and in Supplemental Material VI).

##### Sample 1: KLS

The results from the main analysis suggest that loneliness did not predict smoking initiation (*b* = − 0.17, *SE* = 0.11, *p* = 0.137, hazard ratio (*HR*) = 0.85); see Table 2. This association did not differ between time intervals; see Supplementary Material VII. The likelihood to smoke was found to be statistically significantly higher during period 3 (i.e. from grades 9 to 10) than the mean likelihood to start smoking across all time periods; the likelihood to start smoking during periods 1 (i.e. from grades 7 to 8) and 2 (i.e. from grades 8 to 9) did not differ from the mean. Sex and age were not significantly associated with smoking initiation.

**Table 2:** Discrete time survival analysis for the adolescent samples

	KLS (*N* = 600)					GFT (*N* = 551)^a^					F&H (*N* = 261)				
	*b*	*SE*	*p*	HR	95% CI	*b*	*SE*	*p*	HR	95% CI	*b*	*SE*	*p*	HR	95% CI
Intercept	− **2.49**	**0.10**	**0.001**	**0.08**	[0.07, 0.10]	− **3.06**	**0.28**	**0.001**	**0.04**	**[0.03, 0.08]**	− **1.96**	**0.11**	**0.001**	**0.14**	**[0.11, 0.17]**
Time															
Period 2	− 0.07	0.13	0.581	0.93	[0.71, 1.21]	**0.71**	**0.32**	**0.001**	**2.03**	**[1.08, 3.80]**	− 0.04	0.19	0.797	0.95	[0.66, 1.37]
Period 3	**0.47**	**0.14**	**< 0.001**	**1.61**	**[1.22, 2.12]**	− 1.01	0.52	0.052	0.36	[0.13, 1.01]	0.09	0.20	0.649	1.10	[0.74, 1.62]
Period 4	n.a.	n.a.	n.a.	n.a.	n.a.	0.12	0.52	0.823	1.12	[0.41, 3.11]	0.05	0.22	0.819	1.05	[0.68, 1.62]
Period 5	n.a.	n.a.	n.a.	n.a.	n.a.	− 0.59	0.82	.474	0.55	[0.11, 2.78]	− 0.38	0.27	0.179	0.69	[0.40, 1.19]
Gender															
Female	0.10	0.10	.339	1.10	[0.91, 1.34]	0.06	0.11	0.596	1.06	[0.85, 1.32]	− 0.07	0.09	0.439	0.93	[0.77, 1.12]
Age	0.11	0.10	0.252	1.12	[0.92, 1.35]	**0.42**	**0.11**	**< 0.001**	**1.50**	**[1.23, 1.83]**	0.04	0.09	0.667	1.04	[0.87, 1.24]
Educational type															
Higher general secondary	n.a.	n.a.	n.a.	n.a.	n.a.	− 0.12	0.26	0.640	0.88	[0.53, 1.48]	− 0.06	0.14	.668	0.94	[0.72, 1.24]
Pre−university	n.a.	n.a.	n.a.	n.a.	n.a.	−**0.46**	**0.18**	**0.011**	**0.63**	**[0.44, 0.90]**	− 0.09	0.15	.534	0.91	[0.68, 1.22]
Loneliness	− 0.17	0.11	0.137	0.85	[0.68, 1.05]	− 0.21	0.13	0.116	0.81	[0.63, 1.05]	− 0.18	0.10	0.085	0.84	[0.69, 1.02]

*a Please note that the time interval between the periods varies (period 1 = 1.5 years; Period 2 = 1 year; Period 3 = 0.5 years; Period 4 = 0.5. years; Period 5 = 0.5. years), limiting comparability between the different periods. n.a.* not applicable, *HR* Hazard Ratio, *KLS = Kandinsky Longitudinal Study*, *GFT* = G(F)OOD together, *F&H* = Family & Health study. Categorical variables are sum-to-zero coded. Continuous variables are scaled

##### Sample 2: GFT

The results from the main analysis suggest that loneliness did not predict smoking initiation (*b* = − 0.21, *SE* = 0.13, *p* = 0.116, *HR* = 0.81); see Table 2. This association did not differ between time intervals; see supplemental Table VII. The likelihood of initiating smoking was higher in the second period, compared to the mean of all time periods, but this can be explained by the fact that this period had the longest time interval, with time periods being shorter towards the end of the study. Sex was not significantly associated with smoking initiation. Age was significantly associated with smoking initiation, with older adolescents being more likely to initiate smoking (*b* = 0.42, *SE* = 0.11, *p* = < 0.001, *HR* = 1.50). Furthermore, pre-university students were less likely to initiate smoking compared to the average likelihood across all educational types (*b* = − 0.46, *SE* = 0.18, *p* = 0.011, *HR* = 0.63).

##### Sample 3: F&H

The results from the main analysis suggest that loneliness did not predict smoking initiation (*b* = − 0.18, *SE* = 0.10, *p* = 0.085, *HR* = 0.84); see Table 2. This association did not differ between time intervals; see Supplementary Material VII. The likelihood of initiating smoking did not significantly differ across the time intervals. Other factors, such as sex, age, and education, were not significantly associated with smoking initiation.

Overall, these results show that higher levels of loneliness do not predict a higher or lower chance of starting to smoke.

### University Student Samples

#### Descriptives

In both samples, most participants were female (HSL, 71%; al-RISCO, 66%) (see Table 3). The mean age was 21.73 (HSL) and 22.55 (al-RISCO) years old. The HSL sample consists of only research university students (‘WO’), and the al-RISCO sample consists of 84% research university (‘WO’) and 16% applied university students (‘HBO’). The mean score for loneliness was 1.2 (HSL, range 0–4) and 1.7 (al-RISCO, range 1–3). Across both samples, most participants were non-smokers. In the HSL sample, at T1 to T3, occasional and regular smokers comprised 4.3% and 5.3%, 4.9% and 4.6%, and 3.6% and 3.5% of participants, respectively. For the al-RISCO sample, these figures were 5.9% and 7.9% at T1, 6.2% and 8.4% at T2, and 6.7% and 6.2% at T3, respectively. Smoking quantity (measured in smokers only) in the HSL sample varied between 18.1 and 21.9 cigarettes per week between T1 and T3.

**Table 3:** Descriptive statistics (*n* (%) or M(SD); *n*) for the student samples

	HSL (N = 2,636)	al-RISCO (N = 404)
Gender T1		
Male	576 (23.3%)	137 (33.9%)
Female	1,859 (75.2%)	265 (65.6%)
Other	38 (1.5%)	2 (0.5%)
Age T1	21.73 (2.44); *n = *2636	22.55 (1.86); *n* = 404
Education type T1^a^		
Applied University	n.a.	340 (84.2%)
Research University	2,636 (100%)	64 (15.8%)
Loneliness T1^b^	1.19 (0.73); *n* = 2471	1.72 (0.51); *n* = 404
Smoking status T1		
Non-smoker	1929 (88.5%)	348 (86.1%)
Occasional smoker	113 (5.2%)	24 (5.9%)
Regular smoker	139 (6.4%)	32 (7.9%)
Smoking status T2		
Non-smoker	1751 (87.5%)	318 (84.4%)
Occasional smoker	128 (6.4%)	25 (6.6%)
Regular smoker	122 (6.1%)	34 (9.0%)
Smoking status T3		
Non-smoker	1327 (87.7%)	274 (84.1%)
Occasional smoker	96 (6.3%)	27 (8.3%)
Regular smoker	91 (6.0%)	25 (7.7%)
Smoking quantity (cig/week) in occasional and regular smokers (min = 0; max = 210)		
T1	21.86 (33.78);* n* = 252	n.a.
T2	18.12 (30.00); *n* = 250	n.a.
T3	19.00 (32.01); *n* = 186	n.a.

Descriptive statistics are presented as mean (standard deviation); count for continuous variables and as count (percentage) for categorical variables. *n.a.* not applicable, *HSL = Healthy Student Life project*, *al-RISCO = al-RISCO lifestyle project*

^a^ Higher education in the Netherlands is divided into two types: research (WO) and applied (HBO) universities

^b^ The range for loneliness T1 was 0–4 for HSL and 1–3 for al-RISCO

#### SEM Models: Does Baseline Loneliness Predict Smoking Status at Follow-Up?

##### Sample 4: HSL

The results from the main analysis suggest that baseline loneliness does not predict smoking status at T2 (*β* = 0.05, *SE* = 0.06, *p* = 0.067) but does significantly predict smoking status at T3 (*β* = 0.07, *SE* = 0.06, *p* = 0.035); see Table 4. Smoking status at baseline was consistently positively associated with smoking status at T2 (*β* = 0.67, *SE* = 0.05, *p* < 0.001) and T3 (*β* = 0.60, *SE* = 0.06, *p* < 0.001). Participants who identified as gender other were less likely to smoke frequently at T2 compared to males/females (*β* = − 0.06, *SE* = 0.30, *p* = 0.032). No additional effects were found. In the sensitivity analyses with complete cases/smoking quantity (see Supplementary Material IX.1 and IX.2), no significant effects of baseline loneliness on smoking status/quantity at T3 were found. However, similar trends were observed. The effect of baseline smoking status on smoking status at follow-up remained consistent across the sensitivity analyses. The exploratory analyses (nominal regression) showed that baseline loneliness was specifically associated with being a regular smoker at T3 (OR = 1.62, *p* = 0.033), not an occasional smoker (OR = 1.02, *p* = 0.935, see Supplemental Material VIII). Attrition analyses (Supplemental Material X) revealed that younger participants, participants who felt less lonely, and occasional smokers at baseline were more likely to miss data on smoking status at T2. Additionally, occasional and regular smokers at baseline were more likely to miss data on smoking status at T3.

**Table 4:** Structural equation models for the student samples

T1 variables	HSL (*N* = 2,636)								al-RISCO (*N* = 404)							
	Smoking status T2				Smoking status T3				Smoking status T2				Smoking status T3			
	*b*	β	*SE*	*p*	*b*	β	*SE*	*p*	*b*	β	*SE*	*p*	*b*	β	*SE*	*p*
Gender ^a^	n.a.	n.a.	n.a.	n.a.	n.a.	n.a.	n.a.	n.a.	0.17	0.06	0.26	0.499	0.09	0.03	0.22	0.689
Male vs. female + other ^d^	0.12	0.04	0.10	0.216	0.12	0.04	0.10	0.207	n.a.	n.a.	n.a.	n.a.	n.a.	n.a.	n.a.	n.a.
Other vs. male + female ^d^	− 0.64	− 0.06	0.30	0.032	0.24	0.02	0.19	0.191	n.a.	n.a.	n.a.	n.a.	n.a.	n.a.	n.a.	n.a.
Education level ^b^	n.a.	n.a.	n.a.	n.a.	n.a.	n.a.	n.a.	n.a.	− 0.16	− 0.04	0.25	0.528	0.27	0.08	0.27	− 0.308
Age	− 0.01	− 0.01	0.02	0.729	− 0.02	− 0.04	0.02	0.282	− 0.01	− 0.02	0.05	0.767	0.00	0.00	0.05	− 0.999
Smoking status ^c^	1.82	0.67	0.05	< 0.001	1.50	0.60	0.06	< 0.001	1.51	0.65	0.12	< 0.001	1.34	0.61	0.12	< 0.001
Loneliness	0.10	0.05	0.06	0.67	0.12	0.07	0.06	0.035	− 0.06	− 0.02	0.24	0.812	0.17	0.07	0.20	0.400

^a^Gender was coded as 0 for female and 1 for male

^b^Education level was coded as 1 for applied university (in Dutch ‘HBO’) and 2 for academic university (in Dutch ‘WO')

^c^Smoking status 1 was coded as an ordered factor, with never smokers as 0, occasional smokers as 1, and frequent smokers as 2

^d^Gender at T1 was dummy coded by creating two binary variables: male (1) vs. female + other (0); other (1) vs. male + female (0). *n.a.* not applicable, *HSL* = Healthy Student Life project, *al-RISCO* = al-RISCO lifestyle project

##### Sample 5: al-RISCO Lifestyle Project

Within the al-RISCO sample, baseline loneliness did not predict smoking status at T2 (*β* = − 0.06, SE = − 0.02, *p* = 0.812) or T3 (*β* = 0.17, SE = 0.20, *p* = 0.400), as shown in Table 4. Baseline smoking consistently predicted smoking status at T2 (*β* = 0.65, SE = 0.12, *p* < 0.001), and T3 (*β* = 0.61, SE = 0.12, *p* < 0.001). No additional effects were found. Sensitivity analyses with complete cases confirmed these results (see Supplementary Material IXI.1). The exploratory analyses also showed no significant associations between baseline loneliness and being an occasional or regular smoker at T2 or T3 (see Supplementary Material VIII). It should be noted that the OR between baseline loneliness and being a regular smoker at T3 was 2.48 (in line with the significant result in the HSL sample), but the result was not statistically significant (*p* = 0.073). Attrition analyses (Supplemental Material X) revealed that participants who felt less lonely and frequent smokers at baseline were more likely to miss data on smoking status at T3.

Overall, we did not find strong evidence for a relationship between loneliness and smoking initiation in the adolescent samples or between loneliness and smoking status in the young adult samples. The results of the discrete-time survival analyses in all three adolescent samples suggested that baseline loneliness did not predict smoking initiation. We found evidence in one sample that education level predicted smoking initiation, such that adolescents from higher education levels were less likely to initiate smoking. Further, structural equation models in the two young adult samples did not provide support for the predictive role of loneliness on smoking status. However, we found that baseline loneliness predicted smoking status 18 months later in sample 4. Further analyses revealed that baseline loneliness predicted being a regular smoker at follow-up, but not an occasional smoker at follow-up.

## Discussion

This study aimed to explore the longitudinal associations between loneliness and smoking-related outcomes among adolescents (specifically smoking initiation) and among university students (specifically smoking status). Convergent evidence from our multi-dataset approach did not provide strong support for loneliness predicting smoking initiation in adolescents. Additionally, although one suggestive finding was observed in one of the two samples (for the long-term effect only), no compelling support was found for baseline loneliness predicting later smoking status in university students based on the main analyses. Complementary analyses suggested that loneliness might be associated with being a regular smoker but not an occasional smoker.

### Reflection on Results Adolescent Samples

The findings of the three adolescent samples converged, providing very little support for loneliness predicting smoking initiation in adolescents. Previous studies focusing on longitudinal associations were scarce. The only study that explored the longitudinal relationship between loneliness (in school) and smoking initiation also did not find support for this association using a multivariate analysis approach, but only in univariate analyses (Park, 2009). While other longitudinal (Qualter et al., 2013) and cross-sectional studies have addressed smoking behaviours (Dyal & Valente, 2015; McClure-Thomas et al., 2022; Shadmehr et al., 2019), such as smoking quantity but not initiation, their findings have been mixed. Differences in study designs (e.g. sample characteristics, follow-up periods, cross-sectional designs) and the use of varying smoking measures (e.g. frequency, number of cigarettes, smoking status) limit the comparability of their results with our study.

Interestingly, our study revealed a significant impact of educational type on the likelihood of smoking initiation among adolescents. Specifically, adolescents enrolled in pre-university education programs were less likely to start smoking compared to the average likelihood across all educational types. There could be different explanations for this observation. A study among 1860 adolescents showed that in vocational tracks, popularity norms for smoking and alcohol were more positive and predicted classroom differences in smoking and alcohol (Peeters et al., 2021). In general, attending lower education increases the likelihood of initiating smoking because of limited health literacy (Sørensen et al., 2015).

### Reflection on Results in University Student Samples

The current study did not provide compelling support for baseline loneliness predicting smoking status at 6 and 18 months. However, one suggestive finding was observed in the HSL sample, which showed that baseline loneliness might predict smoking status 18 months later. No previous studies assessing these longitudinal associations in university students were available. Longitudinal studies in (dwelling) older adults do suggest loneliness might predict current smoking (Adebisi et al., 2024; Yang et al., 2022). However, the comparability might be limited given the geographical differences and age of the participants.

The lack of convergence between the two university student samples on baseline loneliness predicting smoking status at 18 months might be explained by the sample size of the HSL project. The association between smoking and loneliness has been proposed to have a small effect size (DeWall & Pond Jr., 2011). Larger samples are more likely to find associations between loneliness and smoking (Dyal & Valente, 2015). This seemed to be the case in the current study: A significant association between loneliness and smoking status was observed in the largest dataset (HSL: *n* = 2636) and not in the smaller one (al-RISCO: *n* = 404), but the effects were in the same direction. Further support for this explanation is that sensitivity analyses with complete cases (i.e. reduced sample size) showed that the significant effect was not observable anymore in the HSL sample. In addition, smoking prevalence in both samples was relatively low (decreasing power); for example, at T1, non-smokers constituted 73%–86% of both samples. Additionally, drop-out rates were acceptable, but attrition analyses for the al-RISCO sample indicated that more frequent smokers were more likely to discontinue participation (see Supplementary Materials X.2). This probably further contributes to limited power in this sample.

To better understand the suggestive finding, we have complemented the main analyses with nominal regression analyses, leading to separate odds ratios for occasional smokers (compared to non-smokers) and regular smokers (compared to non-smokers). Baseline loneliness was associated with being a regular smoker but not an occasional smoker in the HSL dataset. A similar pattern, although not significant, was seen in the al-RISCO dataset. Occasional and regular smokers have different characteristics. In general, regular (daily) smokers score higher on nicotine dependence and smoke for negative reinforcement motives (to avoid withdrawal symptoms) (Mathew et al., 2014). The occasional smokers might smoke more often in social situations, for example, with friends, and for positive reinforcement motives (Oksuz et al., 2007). Therefore, loneliness at baseline could form a higher risk of smoking regularly (coping with loneliness) than occasional smoking (social smoking).

### General Reflection

Several explanations could clarify the limited support for longitudinal associations between loneliness and smoking in both the adolescent and university student samples. One explanation pertains to the underlying motivation for smoking (Dyal & Valente, 2015). Peer influence plays a crucial role in shaping young people’s health behaviour, including smoking (Montgomery et al., 2020). For instance, previous studies suggested that young people may smoke to increase their social acceptance and sense of belonging (Brown et al., 2011; DeWall & Pond Jr., 2011). However, peer influence on motivation to smoke might be particularly relevant in cultures where the behaviour is viewed as a means of gaining popularity. This study focuses on the Netherlands, where smoking rates have been declining since 1999. The decline in smoking rates has also been reflected in the current study as the smoking prevalence at baseline was higher among the adolescents in the F&H study (35.7%; conducted between 2002 and 2003) in comparison to the smoking prevalence at baseline among adolescents in the KLS (4.1%; conducted between 2012 and 2014) and GFT studies (4.2%; conducted between 2017 and 2020). Although smoking among young people remains undoubtedly worrisome, awareness of the health impacts of smoking appears to be effective. This is also reflected in current views on smoking. For example, a Dutch report (*n* = 1008) showed that 80% of youngsters considered smoking as ‘not cool’ any longer and not sociably desirable (Gezondheidsfondsen voor Rookvrij, 2020). To further support this observation, a study of adolescents in the Netherlands from four secondary schools (*n* = 875) concluded that smoking initiation is significantly influenced by friends’ attitudes towards smoking (i.e. what they think or say about it) (Huisman, 2014). Cultural acceptance of smoking, or general lack thereof, among young people in the Netherlands, might explain why the current study did not find strong support for loneliness predicting smoking.

The lack of strong support for loneliness predicting smoking among general adolescent and university student samples, as included in the current study, does not necessarily mean that this is also the case for certain subgroups who are more at risk of experiencing loneliness or initiating smoking. In our samples, the observed levels of loneliness were relatively low, and most participants did not smoke. Regarding subgroups with a higher risk for loneliness: A review by Bayat et al. (2021) indicated that contextual factors like being bullied at school, poor student–teacher relationships, parental divorce, experiencing illness of a close family member, and problematic use of social media were positively associated with loneliness. Alternatively, being non-native and having lower social connection were also positively associated with loneliness (Bayat et al., 2021; Moore et al., 2023), as well as personal characteristics such as shyness, low self-esteem, and poor social skills (Mahon et al., 2006). Regarding subgroups with a higher risk for smoking: Although general views on smoking have changed over the last years (Gezondheidsfondsen voor Rookvrij, 2020), certain subgroups might still smoke to gain social acceptance, and relief from loneliness, for young people who wish to belong to a peer group where smoking is considered ‘cool’. Especially since previous work has acknowledged that young people who have friends who smoke are more likely to initiate smoking themselves (O’Loughlin et al., 2017; Wellman et al., 2016). Hence, future research could examine whether a longitudinal link between loneliness and smoking exists in higher-risk samples, such as those susceptible to peer influence or experiencing poorer mental health. These studies could also control for potential confounders of the link between loneliness and smoking, such as socioeconomic position, depressive symptoms, or stress, as previously suggested by Philip et al. (2022), Dyal and Valente (2015), and Wootton et al. (2021), respectively.

Alternatively, the instruments previously used to assess loneliness might have contributed to the mixed findings from earlier work. Of the six cross-sectional studies supporting a positive association between loneliness and smoking among adolescents in the systematic review by Dyal and Valente (2015), five used a one-item measure of loneliness (including the word ‘lonely’). Marangoni and Ickes (1989) have raised concerns about this, as not everyone recognises themselves as feeling lonely, and others might want to avoid identifying themselves as lonely, given the stigma associated with loneliness (Marangoni & Ickes, 1989). Single-item loneliness measures might assess a specific sub-dimension or variant of loneliness (which might be associated with smoking (initiation)). In contrast, our three adolescent samples used a 12-item scale focusing on loneliness in peer relations, and the two university student samples an 8-item (HSL) or 3-item scale (al-RISCO) reflecting more general feelings of loneliness. Possibly, these instruments capture different aspects of loneliness (which might not be associated with smoking initiation in general adolescent samples or smoking status in university students).

### Strengths and Limitations

In this study, we used a multiple-dataset approach where we answered the same research question within three adolescent and two university student samples. Given that no single study can provide a definite answer to the question of whether longitudinal associations between loneliness and smoking (initiation/status) exist, convergence across multiple datasets provides stronger confidence in our conclusions and provides more robust insights (Hammerton & Munafò, 2021).

Several limitations need to be acknowledged as well. First, the sample sizes varied between the included samples, and although we tried to align the included measures as much as possible, the loneliness and smoking measures were not completely the same for all adolescent or all university student samples. Concerning loneliness, in some samples, brief loneliness scales were used while in others, more comprehensive measures were used. Specifically, among students, the HSL sample used the UCLA scale with eight items, while the al-RISCO sample used the short version with three items (Hughes et al., 2021; Russell et al., 1980). Although both scales exhibit acceptable psychometric properties, the 8-item version tends to have better psychometric properties compared to the 3-item version, offering a more reliable measure of loneliness (Lin, 2022). Consequently, the slightly different measures might limit the compatibility of the findings. However, the scales are conceptually comparable overall, as all capture the subjective experience of loneliness (Maes et al., 2022). Also, previous research has shown a high degree of convergence between RULS-8 and the original UCLA-Revised scale (*r* = 0.92), highlighting that both scales assess the same underlying construct (Goossens et al., 2014). Concerning smoking measures, we used different measures per examined developmental stage. For adolescents, we zoomed in on smoking initiation, given that at this stage, most adolescents have not yet progressed to regular smoking. For young adults, we zoomed in on regular smoking, given that at this stage, most young adults have initiated or experimented with smoking. The use of different measures across the utilized samples might cause discrepancies and reduce the comparability of the findings across the samples. However, we do believe this impact is limited, given the high level of observed convergence in findings across the samples.

Additionally, for university students, we used a categorical outcome with three categories to assess smoking status. Combined with the low prevalence of smokers, this may have limited the statistical power of our analyses. Another methodological limitation involves the time intervals between surveys for one of the adolescent samples (GFT), which varied and potentially affected the detection of smoking initiation. Future studies should carefully consider the time scale at which the process (of loneliness leading to smoking initiation) would take place. Also, it is important to note that the young adult samples in our study were imbalanced in favour of women, and our findings are, thus, generalizable with gender constraints. Future studies should strive for more gender-balanced young adult samples.

Another possible limitation is that we used datasets collected in schools and universities in the Netherlands specifically. Thus, our results may not be generalizable in cultures that have different social norms and societal perceptions. Future studies can test if socio-cultural factors play a role in the relation between loneliness and smoking, and compare the findings across cultures.

Moreover, reliance on self-reported data for smoking and loneliness introduces the risk of social desirability bias and recall inaccuracies (Grimm, 2010), which can affect the validity of the findings. In line with this, attrition analyses among the young adult samples showed that variables like loneliness, smoking status, and age (only sample 4) were linked to a higher risk of dropping out (see Supplemental Material X). While it is difficult to explicitly distinguish between missing at random (MAR) and missing not at random (MNAR) missingness patterns (Hughes et al., 2021; Little et al., 2014), the findings of the attrition analyses suggest that missingness may have depended on prior loneliness or smoking scores. This could have led to underreporting of loneliness and smoking, and potentially, biased findings (Hughes et al., 2021; Little et al., 2014). Specifically, when individuals with more feelings of loneliness or individuals who smoke were more likely to drop out, averages and associations involving these variables may be attenuated due to the underrepresentation of higher-risk individuals (Weuve et al., 2012). Additionally, this could reduce the generalizability of the findings, as the sample might not have been fully representative of the general student population in the Netherlands. Future studies should be mindful of missing data patterns. Statistical procedures such as Bayesian Models or Heckman Selection Models can help to account for missing data patterns (Galimard et al., 2018; Linero & Daniels, 2018). It is also important to clarify what constitutes smoking initiation. While one puff of a cigarette may not fully equate to smoking initiation, it is often considered the beginning of experimenting with smoking. Consequently, a more robust measure, such as smoking at least one whole cigarette, may more accurately define smoking initiation.

Furthermore, the COVID-19 pandemic covered some periods of the study for samples such as from GFT, HSL, and al-RISCO, which might have affected loneliness experiences and smoking behaviours. However, the latter might not be affected to a high extent, as previous work among university students indicated that smoking behaviours remained stable when comparing before and during the first COVID-19 lockdown (van Hooijdonk et al., 2022). In contrast, several reviews have indicated that mental health problems as well as loneliness increased among younger people during the COVID-19 pandemic (Ernst et al., 2022; Pai & Vella, 2021; Saulle et al., 2022).

Last, in the current study, we focused on assessing whether loneliness predicted smoking, while the temporal relationship could also occur in the other direction (smoking predicting more loneliness). The relationship between these variables could be reciprocal, with loneliness influencing smoking behaviour and smoking behaviour potentially exacerbating feelings of loneliness (Wootton et al., 2021).

### Conclusions and Implications for Adolescents and University Students

Overall findings from our multi-dataset approach did not provide compelling support for (baseline) loneliness predicting smoking initiation (among adolescents) or smoking status (among university students). Complementary analyses in the student samples suggested that baseline loneliness might be associated with later regular smoking but not occasional smoking. Future studies could focus on exploring longitudinal associations in higher-risk groups for either loneliness or smoking, such as those susceptible to peer influence or experiencing poorer mental health. Our findings contribute to the limited longitudinal research on the association(s) between loneliness and smoking (Dyal & Valente, 2015). However, more longitudinal research with larger sample sizes is needed to make stronger claims.

## Supplementary Information

Below is the link to the electronic supplementary material.

ESM 1 (DOCX 216 KB)

## Data Availability

Data are available on request (contact person: Jacqueline Vink; jacqueline.vink@ru.nl). All datasets can be requested through the Radboud Data Repository: https://doi.org/10.34973/x37c-f898. R codes have been made available via OSF: https://osf.io/4sk5h/.
