# Supplementary material for: Longitudinal Examination of Loneliness and Smoking in Adolescents and Young Adults: A Multiple Dataset Study
Source: Int J Ment Health Addict. 2025 Aug 5;24(4):2827–49. doi: 10.1007/s11469-025-01522-1 (PMC13499753; doi:10.1007/s11469-025-01522-1)
Supplement: Supplementary file 1 [file 11469_2025_1522_MOESM1_ESM.docx]

**Supplementary Materials**

**Content**

I. Deviations from pre-registration
II. Detailed sample descriptions
III. Detailed information on used materials and coding
IV. Methods sensitivity analyses
V. Methods attrition analyses
VI. Results smoking life table for adolescent samples.
VII. Results discrete time survival analysis including time x loneliness
VIII. Results exploratory analyses in the student samples
IX. Results sensitivity analyses
X. Results attrition analyses
I. **Deviations from pre-registration**

Deviations from the pre-registration have been reported below:

- In the structural equation model of the sensitivity analyses on HSL data (sample 4), we excluded participants with gender ‘other’ because several warnings were encountered while running the analyses (probably due to the small number of participants with gender ‘other’).
- In the main analyses for students (samples 4 and 5) smoking status (non-smoker, occasional smoker, regular smoker) is considered an ordered variable (resulting in one beta). To better understand possible contrasts between the different smoking status groups, we have performed exploratory analyses. We complemented the main analyses with two multinominal logistic regression analyses for each sample. The first exploratory analyses included smoking status (non-smoker, occasional smoker, regular smoker) at W2 as the dependent variable and the second exploratory analyses included smoking status at W3 as the dependent variable. The independent variables were the same as in the main analyses (gender, educational level (al-RISCO only), smoking status W1, loneliness W1). With these exploratory analyses, the outcome variable was given more than two categories, without order, which allowed to estimate different betas for occasional and regular smokers (compared to the reference group of non-smokers).

II. **Detailed sample descriptions**

***Study 1: Kandinsky Longitudinal Study (KLS)***

The KLS (<https://osf.io/vdhnp/>) is a longitudinal study designed to detect youths at risk for social and emotional problems in secondary education in The Netherlands. This ongoing study started in 2010. Since then, the social-emotional functioning of all students in Grades 7 through 10 (i.e., the first four years of secondary education in The Netherlands) is assessed annually. In this study, we will use the data collected between 2012-2016.

Each year, the head of the school requested an assessment of students’ socio-emotional well-being and claimed responsibility for the parental consent procedure. Schools distributed a letter among the parents in which the purpose and procedures of the assessment were described. Until 2018 (thus, during the waves that are used in the current study), a passive parental consent procedure was used. Parents were informed about the study via a letter and asked to respond if they wanted to exclude their child from participation. Adolescents assented at the start of the assessment. Anonymized data was made available to the researchers for scientific purposes.

At each wave, participants were asked to complete a computerized survey on individual computers during a 45 to 60-minute classroom session. At least two researchers were present during the assessment in the classroom. Before the assessment, one researcher explained the goal and set-up of the study. Participants were also told that the data would be processed anonymously and handled confidentially. Adolescents were asked to keep their answers to themselves and to be truthful in answering all questions. During the data collection, adolescents sat at a private desk with partitioning screens. They were not allowed to talk to each other during the assessment but could ask questions to the researchers. They could stop at any given moment. At the end of the session, they received a small gift. Moreover, they automatically participated in a raffle where they could win a tablet.

The study procedures were approved by the Institutional Review Board of Radboud University, Nijmegen, The Netherlands (ECG2012-2505-038).

***Study 2: “G(F)OOD together” study (GFT)***

The “G(F)OOD together!” research project is a 6-wave longitudinal observational study on Dutch adolescents’ and their parents’ health behaviours (van den Broek et al., 2020; 2022; <https://osf.io/bysgq/>). Wave 1 to 3 took place before the COVID-19 pandemic (fall 2017, spring 2018, and spring 2019), and Wave 4 to Wave 6 took place during the COVID-19 pandemic (i.e., fall 2020, spring 2021, and fall 2021; see Figure 1). Furthermore, at Wave 4, adolescents were asked to retrospectively report on their experiences and behaviours during the first Dutch lockdown (Wave 4 retro, Spring 2020). Adolescents were recruited through seven secondary schools in the South and the East of the Netherlands. Adolescents’ parents were also invited to participate in the research project, but their data will not be used in the current study. In Wave 5, *n* = 17 additional 16-to-18-year-old adolescents and one of their parents were recruited to take part in the research project through Facebook advertisements, to increase the sample size.

The study originally included four waves. An active parental consent procedure was used, in which a letter describing the longitudinal study was mailed to the parents of the adolescents. Parents were asked to return a consent form indicating whether they agreed to their child’s participation in the study. Subsequently, adolescents were asked to provide consent to participate in the study themselves as well. To assess the impact of the COVID-19 pandemic, consent to participate in the study was asked again at Wave 5, for participation in Waves 5 and 6.

At the onset of the study, participants were informed that participation was voluntary, that answers would be processed anonymously, and that they could withdraw from the study at any moment. At all waves, adolescents completed an online survey. Surveys were administered through Qualtrics Survey Software on small laptops. At Waves 1 to 3, adolescents completed an online survey at school during one classroom hour (approximately 50 minutes). At Waves 4 to 6, due to the restrictions imposed during the COVID-19 pandemic, adolescents completed an online survey on their own device without the researchers visiting the schools. At Waves 1 to 4, adolescents received a small gift for their participation, and thirty-four gift vouchers (values: 5 to 50 euros) and three weekend getaways (value: 250 euros) were raffled among the participating families. Furthermore, all adolescents received a 5 euros voucher at Wave 5 and a 10 euros voucher at Wave 6 to thank them for their participation.

The study procedures were approved by the Ethics Committee Social Sciences of Radboud University, Nijmegen, The Netherlands (ECSW-2017-0805-516 for Waves 1 to 4; amendment ECSW-2020-122 for online administration of adolescent surveys Wave 4; ECSW-2020-182 for Waves 5 to 6; and amendment ECSW-2021-043 for online recruitment of additional participants Wave 5).

*Figure 1.* Timeline of the G(F)OOD together study; W = Wave.

***Study 3: Family and Health study (F&H)***

The F&H study was designed to investigate socialization processes underlying health behaviors (Harakeh et al., 2005). Approximately 5400 families with at least two children aged 13-16 years from 22 municipalities in the Netherlands were approached to participate in this project. The families received a letter in which they were asked to return the included consent response form, if they were willing to participate. A total of 885 families consented to participate and were contacted via phone to assess eligibility. Families were eligible if: 1. Parents were married or lived together, 2. All family members were biologically related, 3. Siblings were not twins and 4. Offspring did not have mental of physical disabilities. A total of 765 families met the eligibility criteria, but because of financial constraints, this number was reduced to 428 Dutch families. Families were selected such that there was an equal division of education and an equal amount of sibling dyads (boy-boy, boy-girl, girl-boy, girl-girl).

The F&H study included 6 waves of data collection over 5 years. Baseline (Wave 1) data was collected between November 2002 and April 2003. Participating families at other waves were: 416 (Wave 2), 404 (Wave 3), 356 (Wave 4), 326 (Wave 5), and 313 (Wave 6). At baseline, all families were visited at their homes and family members were asked to complete surveys individually. At subsequent waves, most of the families were again visited at their homes, but due to practical and financial reasons, some families received the surveys via post (8-25% per wave).

Approval for the study was obtained from the Central Committee on Research Involving Human Subjects.

***Study 4: Healthy Student Life project (HSL)***

The HSL project (van Hooijdonk et al., 2023; <https://osf.io/e4d5b/>) is an ongoing longitudinal survey study designed to investigate the well-being, lifestyle, individual and contextual factors of university students at Radboud University (Research University in Nijmegen, the Netherlands). So far, three waves of survey data have been collected (Wave 1 October-November 2021, Wave 2 May-July 2022, and Wave 3 May-July 2023).

At each wave, no exclusion criteria were applied during recruitment, and all students at that moment enrolled at Radboud University (±25.000) were invited (regardless of their participation in previous waves) to complete an online survey in Qualtrics. This invitation was sent from the Radboud University mail account to the institutional email addresses of the students. Student were asked to give active consent before they completed their first survey. At Wave 3, a similar survey was sent to Wave 2 participants who indicated at Wave 2 to be willing to join follow-up research after their graduation and who were not registered as a student anymore at Wave 3 (*n* invited = 484). We refer to this data collection as Wave 3 alumni.

At each wave, the survey (administered through Qualtrics Survey Software) consisted of several main topics: 1) well-being, 2) lifestyle, 3) personal factors, and 4) contextual/university-related factors. The Wave 3 alumni survey also included some questions on the transition from studying to working. On average, completing the survey would take ±20-30 minutes. Participants joined voluntarily and could decide to stop their participation at any time during the study. It was also possible to take breaks during the completion of the survey. At all waves, different gift vouchers (for a weekend trip, online store, or campus sports centre) were raffled off among participants who finished the survey, and at Wave 3, all participants (excluding Wave 3 alumni) received a free cup of coffee from a local campus supermarket upon completing the survey. During all data collections, several recruitment activities were used to increase the response, including messages on the Radboud University and faculty social media channels, distribution of sustainable postcards, notifications in the student newsletter, and the distribution of recruitment materials during teaching activities (e.g., lectures or practical sessions).

Data collection of the HSL project has been approved by the Ethics Committee Social Sciences of Radboud University (ECSW-2021-086).

***Study 5: al-RISCO lifestyle project (al-RISCO)***

The al-RISCO project is a 5-wave longitudinal survey study that examines the effects of cognitive, social, and emotional factors on student lifestyles (Rubio et al., 2024; <https://osf.io/urhdt/>). It focuses specifically on the alcohol use of university students as they transition from academic settings to life after graduation. Dutch applied and research university students were invited to take part in a two-year-long online survey study with five waves of data. The project started in early 2021 and was completed by May 2023. For the current study, three waves of data were selected. In this study, we refer to these waves as Wave 1 (February-March 2021), Wave 2 (September-November 2021), and Wave 3 (September-November 2022: see Figure 2). Each wave of the survey took approximately 30 to 45 minutes to complete.

Various recruitment methods were used, including social media advertisements, distributing flyers in student-related venues, contacting university organizations, utilizing the participant recruitment system of the university, and snowball sampling. Interested individuals completed a brief online screening and were entered into a lottery for coupons worth 50 euros, redeemable at a popular Dutch web shop. For each of the four measures completed, participants received a 10-euro coupon and a newsletter with (preliminary) sample descriptions and other study communications.

To be eligible to participate in the al-RISCO project, participants had to fulfil certain criteria: 1) Being an applied or research university student in the last year of their bachelor studies or any year of their master studies. 2) Being a student from a Dutch research or applied university with a good command of the Dutch language. It was important that our sample was representative of university students engaged in the Dutch cultural context, as this might influence drinking behaviour as well as lifestyle in general. 3) Engaging in heavy (episodic) drinking during the month prior to the COVID-19 outbreak. Heavy drinking behaviour was asked retrospectively during the screening. We aimed to include those students who were engaging in the behaviour during the “regular” student context, before the outbreak started. Heavy episodic drinking was operationalized as drinking at least once a week, six or more drinks daily for men and four or more drinks daily for women, while the criteria for those who did not identify as either men or women was five or more drinks. This criterion was selected based on heavy drinking (in Dutch: zwaar drinken) guidelines in the Netherlands (Trimbos Institute, 2017).

A total of 1,176 students were initially screened during the fall semester of 2020. Out of these, 634 students were classified as heavy drinkers and were invited to participate; 443 (70%) agreed to participate after reading and accepting the letter of consent before the online survey and completed the baseline measure (Wave 1) in mid-February 2021. Retention rates of the 443 participants across the follow-up measures were very high: 95%, 90%, 84%, and 87%, for Waves 2 to 5, respectively. A final sample of 439 participants (Mage at baseline = 22.5 years, SD = 1.8 years; 66% women) was analysed after excluding 4 individuals due to technical issues (n = 1) and needing help for drinking problems (n = 3).

Data collection for the al-RISCO lifestyle project has been approved by the Ethics Committee Social Sciences of Radboud University (ECSW-2020-059).

Figure 2. Visualization of the al-RISCO study timeline and COVID-19 related restrictions during data collection.

III. **Detailed information on used materials and coding**

***Study 1: Kandinsky Longitudinal Study (KLS)***

**Loneliness:** The Loneliness and Aloneness Scale for Children and Adolescents (LACA)- subscale ‘loneliness in relation to peers was used to assess loneliness (Goossens & Maes, 2020). The scale consists of 12 items, example item “I feel abandoned by my friends”. For each of the 12 items, participants were asked to indicate how often the following feelings concerned them on a 4-point scale ranging from 0 = Never to 3 = Often. An average score (range 0-3) was calculated for the 12 items. Cronbach’s alpha for Wave 1-4 varied between .89 and .92. Higher scores indicate more loneliness.

**Smoking initiation:** To assess smoking initiation, in all waves, adolescents were asked to indicate which of the following statements fitted them best: “1. I have never smoked, not even a puff”; “2. I did try smoking, but I don't smoke anymore”; “3. I quit, I always smoked less than once a week”, “4. I quit, after smoking at least once a week for a time” “5. I try smoking every now and then”; “6. I smoke less than once a month”, “7. I do not smoke weekly, but at least once a month”, “8. I do not smoke daily, but at least once a week”, and “9. I smoke at least once a day”. All participants in the analytic sample have never smoked at baseline. For each following wave, we checked whether they indicated that they had ever smoked. If participants indicated that they had never smoked (statement 1), smoking initiation was coded as 0 (No). Once a participant indicated that they had ever smoked (any of the statements 2-9) smoking initiation was coded at 1 (Yes), and the following waves of data were removed from the dataset for that participant.

**Biological Sex:** Participants were asked, “Are you a boy or a girl?”. Answer options included: (0) Boy and (1) Girl.

**Age:** Participants’ age at Wave 1 was calculated based on their date of birth and date of participation (registered when completing the survey).

**Educational type:** Not applicable for the KSL sample (information was not available).

***Study 2: “G(F)OOD together” study (GFT)***

**Loneliness:** The Loneliness and Aloneness Scale for Children and Adolescents (LACA)- subscale ‘loneliness in relation to peers was used to assess loneliness (Goossens & Maes, 2020). The scale consists of 12 items, example item “I feel abandoned by my friends”. For each of the 12 items, participants were asked to indicate how often the following feelings concerned them on a 4-point scale ranging from 0 = Never to 3 = Often. An average score (range 0-3) was calculated of the 12 items. Cronbach’s alpha for Wave 1-5 varied between .86 and .92. Higher scores indicate more loneliness.

**Smoking initiation:** To assess smoking initiation, in all waves, adolescents were asked to indicate which of the following statements fitted them best: “1. I have never smoked”, “2. I took a puff of a cigarette a few times”, “3.	I used to smoke (regularly), but quit smoking now”, “4. I smoke once a week or less”, “5. I smoke several times a week”, and “6.I smoke daily”. All participants in the analytic sample have never smoked at baseline. For each following wave, we checked whether they indicated that they had ever smoked. If participants indicated that they had never smoked (statement 1), smoking initiation was coded as 0 (No). Once a participant indicated that they had ever smoked (any of the statements 2-6) smoking initiation was coded as 1 (Yes), and the following waves of data were removed from the dataset for that participant.

**Biological Sex:** Participants were asked to respond to the question “I am a …”. Answer options included: (0) Boy and (1) Girl.

**Age:** Participants’ age at Wave 1 was calculated based on their date of birth and date of participation (registered when completing the survey).

**Educational type:** Educational level was provided by the schools, categorized as (2) pre-vocational education, (2) higher general secondary education, and (3) pre-university education.

***Study 3: Family and Health study (F&H)***

**Loneliness:** The Loneliness and Aloneness Scale for Children and Adolescents (LACA)- subscale ‘loneliness in relation to peers was used to assess loneliness (Goossens & Maes, 2020). The scale consists of 12 items, example item “I feel abandoned by my friends”. For each of the 12 items, participants were asked to indicate how often the following feelings concerned them on a 4-point scale ranging from 0 = Never to 3 = Often. An average score (range 0-3) was calculated of the 12 items. Cronbach’s alpha for Wave 1-5 varied between .91 and .94. Higher scores indicate more loneliness.

**Smoking initiation:** To assess smoking initiation, in all waves, adolescents were asked to indicate which of the following statements fitted them best: “1. I have never smoked, not even a puff”; “2. I did try smoking, but I don't smoke anymore”; “3. I quit, I always smoked less than once a week”, “4. I quit, after smoking at least once a week for a time” “5. I try smoking every now and then”; “6. I smoke less than once a month”, “7. I do not smoke weekly, but at least once a month”, “8. I do not smoke daily, but at least once a week”, and “9. I smoke at least once a day”. All participants in the analytic sample have never smoked at baseline. For each following wave, we will check whether they indicated that they had ever smoked. If participants indicated that they had never smoked, smoking initiation will be coded as 0 (No). Once a participant indicated any other statement than “I have never smoked” as best fitting, smoking initiation will be coded at 1 (Yes), and the following waves of data will be removed from the dataset for that participant.

**Biological Sex:** Participants were asked to select their sex. Answer options included: (0) Boy and (1) Girl.

**Age:** Participants’ age at Wave 1 is calculated by subtracting their birthdate from the date of data collection.

**Educational type:** Educational level will be recoded into the following categories: (1) Pre-vocational secondary education, (2) Higher general secondary education, (3) Pre-university education.

***Study 4: Healthy Student Life project (HSL)***

**Loneliness:** The Roberts UCLA Loneliness Scale (RULS-8) was used to assess loneliness (Goossens et al., 2014). This scale consists of 8 items, example item: “I feel in tune with people around me”. For each of the 8 items, participants were asked to indicate how correct that statement was to them on a 5-point scale ranging from 0 = Completely incorrect to 4 = Completely correct. A mean score of the RULS-8 was calculated (range 0-4) for all participants who answered at least 80% of the items. Accordingly, positively framed items will be reversely coded. Higher scores indicate more loneliness.

**Smoking status:** All participants were classified according to their smoking status. We used the question “How often did you smoke cigarettes or shag in the past half year?”. The answer categories on this question included (1) Never, (2) Ever taken a puff of a cigarette a few times, (3) Ever smoked (regularly), but not in the past half year, (4) Monthly or less, (5) 2 to 4 times a month, (6) 2 to 3 times a week, (7) 4 to 5 times a week and (8) 6 to 7 times per week. Participants who answered (1), (2), or (3) were coded as smoking status “non-smoker” (0). Participants who answered (4) or (5) were coded as smoking status “occasional smoker” (1). Participants who answered (6), (7), or (8) were coded as smoking status “regular smoker” (2).

**Smoking quantity (sensitivity analysis):** For all participants, we calculated their smoking quantity in cigarettes/week. We used the question “How often did you smoke cigarettes or shag in the past half year?” for this. The answer categories on this question included (1) Never, (2) Ever taken a puff of a cigarette a few times, (3) Ever smoked (regularly), but not in the past half year, (4) Monthly or less, (5) 2 to 4 times a month, (6) 2 to 3 times a week, (7) 4 to 5 times a week and (8) 6 to 7 times per week. Participants who answered (1), (2), or (3) were coded a smoking quantity of 0 cigarettes/week. Participants who answered (4) and (5) received a follow-up to assess how many cigarettes they smoked on average per month. For these participants smoking quantity of cigarettes/week was calculated by dividing the number of cigarettes they smoked per month by 4,3 (weeks per month; 52 weeks per year divided by 12 months). Participants who answered (6) or (7) received a follow-up question to assess how many cigarettes they smoked on average per week. For these participants, the answer to this question was used as their smoking quantity in cigarettes/week. Participants who answered (8) received a follow-up question to assess how many cigarettes they smoked on average per day. For these participants smoking quantity in cigarettes/week was calculated by multiplying the number of cigarettes they smoked per day by 7 (days per week).

**Gender:** Participants were asked, “What is your gender?”. Answer options included: (1) Male, (2) Female or (3) Other.

**Age:** Participants' age at Wave 1was calculated based on their date of birth (request via administrative systems of the university) and date of participation (registered when completing the survey). In case participants' age at W1 was not available (for example because participants joined the study at one of the follow-up waves), age at Wave 1 was estimated based on data of participation in the next available wave.

**Educational type:** Not applicable for the HSL sample as all students were from the same educational type (research university).

***Study 5: al-RISCO lifestyle project (al-RISCO)***

**Loneliness:** The Three-Item Loneliness Scale (UCLA-revised) was used to assess loneliness (Hughes et al., 2021; Russell et al., 1980). This scale consists of 3 items, example item: “How often do you feel you lack companionship?”. For each of the 3 items, participants were asked to indicate how often the following feelings concerned them on a 3-point scale ranging from 1= Hardly ever to 3 = Often. A mean score of the UCLA-revised was calculated (range 1-3) for participants who completed the whole scale. Higher scores indicate more loneliness.

**Smoking status:** All participants were classified according to their smoking status. We used the question “Which statement about smoking is most applicable to you?”. The answer categories on this question included (1) I have never smoked, (2) I have taken a puff of a cigarette a few times, (3) I used to smoke (regularly), but quit smoking now, (4) I smoke once a week or less, (5) I smoke several times a week, but not daily and (6) I smoke daily. Participants who answered (1), (2), or (3) were coded as smoking status “non-smoker” (0). Participants who answered (4) were coded as smoking status “occasional smoker” (1). Participants who answered (5) or (6) were coded as smoking status “regular smoker” (2).

**Gender:** Participants were asked, “Which sex do you identify with most?”. Answer options included: (1) Male, (2) Female or (3) Diverse.

**Age:** Participants were asked, “What is your age?”.

**Educational type:** Participants were asked, “What is your educational level?”. Answer options included (1) I am following/followed a research program or (2) I am following/followed an applied program.

IV. **Methods sensitivity analyses**

*Adolescent samples (Samples 1, 2 and 3)*

In accordance with the pre-registration, no sensitivity analyses were performed.

*Student Samples (Samples 4 and 5)*

In both samples, the main analysis was repeated in complete cases only. Additionally, in the HSL sample, a linear regression analysis with SEM was performed including were smoking status (T1, T2 and T3) was replaced by the continuous variable smoking quantity (T1, T2 and T3). The Full Information Likelihood (FIML) estimator was used to deal with missing data and the Maximum Likelihood with Robust standard errors (MLR) estimator was used to adjust for non-normality in the data (Graham, 2009).

V. **Methods attrition analyses**

*Adolescent samples (Samples 1, 2 and 3)*

In accordance with the pre-registration, we did not perform attrition analyses for the three adolescent samples (due to the nature of the discrete-time analysis).

*Student Samples (Samples 4 and 5)*

In both samples, two logistic regression analyses were performed to investigate missing data patterns. The “glm” function in R with a binomial family, suitable for logistic outcomes was used accordingly. All T1 variables (loneliness, smoking status, smoking quantity (HSL only), gender, age, and education level (al-RISCO only)) were used as predictors, and missing data on smoking status at 1) T1 (yes or no) or 2) T2 (yes or no) was included as the dependent variable.

VII. **Results smoking life table for adolescent samples**

|  | KLS (*N* = 600) | | | |  | GFT (*N* = 551)^a^ | | | |  | F&H (*N* = 261) | | | |
| --- | --- | --- | --- | --- | --- | --- | --- | --- | --- | --- | --- | --- | --- | --- |
|  | Total | Smoking Initiation | Censored | HR |  | Total | Smoking Initiation | Censored | HR |  | Total | Smoking Initiation | Censored | HR |
| Period 1 (T1→T2) | 600 | 33 | 41 | .055 |  | 551 | 60 | 288 | .109 |  | 261 | 45 | 7 | 0.17 |
| Period 2 (T2→T3) | 526 | 40 | 237 | .076 |  | 203 | 19 | 0 | .094 |  | 209 | 29 | 31 | 0.14 |
| Period 3 (T3→T4) | 249 | 32 | 217 | .129 |  | 184 | 3 | 117 | .016 |  | 149 | 21 | 9 | 0.14 |
| Period 4 (T4→T5) | n.a. | n.a. | n.a. | n.a. |  | 64 | 3 | 11 | .047 |  | 119 | 16 | 5 | 0.13 |
| Period 5 (T5→T6) | n.a. | n.a. | n.a. | n.a. |  | 50 | 1 | 49 | .020 |  | 98 | 9 | 89 | 0.09 |

*Note.* n.a. = not applicable, HR = Hazard Ratio, KLS = Kandinsky Longitudinal Study, GFT = G(F)OOD together, F&H = Family & Health study. ^a^ Please note that the time interval between the periods varies (period 1 = 1.5 years; Period 2 = 1 year; Period 3 = 0.5 years; Period 4 = 0.5. years; Period 5 = 0.5. years), limiting comparability between the different periods.

VIII. **Results discrete-time survival analysis including time x loneliness for the adolescent samples**

|  | KLS (*N* = 600) | | | | |  | GFT (*N* = 551) | | | | |  | F&H (*N* = 261) | | | | |
| --- | --- | --- | --- | --- | --- | --- | --- | --- | --- | --- | --- | --- | --- | --- | --- | --- | --- |
|  | *b* | *SE* | *p* | HR | 95% CI |  | *b* | *SE* | *p* | HR | 95% CI |  | *b* | *SE* | *p* | HR | 95% CI |
| Intercept | -2.48 | 0.10 | < .001 | 0.08 | [0.07, 0.10] |  | **-3.18** | **0.32** | **< .001** | **0.04** | **[0.02, 0.07]** |  | **-1.97** | **0.11** | **< .001** | **0.14** | **[0.11, 0.17]** |
| Time |  |  |  |  |  |  |  |  |  |  |  |  |  |  |  |  |  |
| Period 2 | -0.07 | 0.14 | .592 | 0.93 | [0.71, 1.21] |  | **0.82** | **0.36** | **.023** | **2.31** | **[1.13, 4.73]** |  | -0.04 | 0.19 | .848 | 0.97 | [0.67, 1.40] |
| Period 3 | **0.48** | **0.14** | **< .001** | **1.62** | **[1.22, 2.15]** |  | -1.24 | 0.73 | .091 | 0.29 | [0.07, 1.23] |  | 0.03 | 0.21 | .876 | 1.02 | [0.67, 1.56] |
| Period 4 | n.a. | n.a. | n.a. | n.a. | n.a. |  | 0.09 | 0.61 | .880 | 1.09 | [0.32, 3.69] |  | 0.05 | 0.22 | .814 | 1.05 | [0.68, 1.63] |
| Period 5 | n.a. | n.a. | n.a. | n.a. | n.a. |  | -0.54 | 0.90 | .549 | 0.59 | [0.10, 3.43] |  | -0.36 | 0.28 | .205 | 0.71 | [0.41, 1.22] |
| Sex |  |  |  |  |  |  |  |  |  |  |  |  |  |  |  |  |  |
| Girls | 0.09 | 0.10 | .345 | 1.10 | [0.90, 1.33] |  | 0.05 | 0.11 | .636 | 1.05 | [0.85, 1.31] |  | -0.08 | 0.09 | .419 | 0.93 | [0.77, 1.11] |
| Age | 0.11 | 0.10 | .256 | 1.12 | [0.92, 1.35] |  | **0.43** | **0.11** | **< .001** | **1.51** | **[1.24, 1.85]** |  | 0.04 | 0.09 | .683 | 1.04 | [0.87, 1.24] |
| Educational type |  |  |  |  |  |  |  |  |  |  |  |  |  |  |  |  |  |
| Higher general secondary | n.a. | n.a. | n.a. | n.a. | n.a. |  | -0.13 | 0.27 | .618 | 0.88 | [0.52, 1.47] |  | -0.06 | 0.14 | .685 | 0.95 | [0.72, 1.24] |
| Pre-university | n.a. | n.a. | n.a. | n.a. | n.a. |  | **-0.46** | **0.18** | **.011** | **0.63** | **[0.45, 0.90]** |  | -0.11 | 0.15 | .471 | 0.90 | [0.67, 1.20] |
| Loneliness | -0.17 | 0.12 | .149 | 0.85 | [0.67, 1.06] |  | -0.50 | -1.32 | .187 | 0.61 | [0.29, 1.27] |  | -0.21 | 0.11 | .064 | 0.81 | [0.64, 1.01] |
| Time x Loneliness |  |  |  |  |  |  |  |  |  |  |  |  |  |  |  |  |  |
| Period 2 x Loneliness | 0.02 | 0.15 | .877 | 1.02 | [0.76, 1.38] |  | 0.66 | 0.41 | .11 | 1.91 | [0.87, 4.16] |  | 0.06 | 0.20 | .786 | 1.06 | [0.71, 1.58] |
| Period 3 x Loneliness | 0.05 | 0.17 | .791 | 1.05 | [0.75, 1.47] |  | -0.85 | 1.05 | .42 | 0.44 | [0.06, 3.31] |  | -0.31 | 0.25 | .211 | 0.73 | [0.45, 1.19] |
| Period 4 x Loneliness | n.a. | n.a. | n.a. | n.a. | n.a. |  | -0.41 | 0.94 | .67 | 0.67 | [0.11, 4.12] |  | -0.07 | 0.24 | .769 | 0.93 | [0.58, 1.49] |
| Period 5 x Loneliness | n.a. | n.a. | n.a. | n.a. | n.a. |  | 0.45 | 0.75 | .55 | 1.56 | [0.37, 6.55] |  | 0.14 | 0.27 | .620 | 1.15 | [0.67, 1.96] |

*Note.* n.a. = not applicable, HR = Hazard Ratio, KLS = Kandinsky Longitudinal Study, GFT = G(F)OOD together, F&H = Family & Health study. Categorical variables are sum-to-zero coded. Continuous variables are scaled.

IX. **Results exploratory analyses in the student samples**

*Exploratory analyses: nominal regression analyses in the student samples HSL and al-RISCO*

|  | HSL T1-T2 (*N* = 1,727) | | | | | | | |  | HSL T1-T3 (*N* = 1,300) | | | | | | | |
| --- | --- | --- | --- | --- | --- | --- | --- | --- | --- | --- | --- | --- | --- | --- | --- | --- | --- |
|  | Occasional smoker T2 | | | | Regular smoker T2 | | | |  | Occasional smoker T2 | | | | Regular smoker T2 | | | |
|  | *B* | OR | *SE* | *p* | *B* | OR | *SE* | *p* |  | *B* | OR | *SE* | *p* | *B* | OR | *SE* | *p* |
| Gender Male^a^ | 0.46 | 1.58 | 0.32 | .154 | **0.79** | **2.20** | **0.38** | **.036** |  | 0.31 | 1.36 | 0.34 | .368 | 0.30 | 1.34 | 0.40 | .460 |
| Gender Other^a^ | -0.21 | 0.81 | 1.16 | .856 | -0.81 | 0.45 | 0.46 | .586 |  | **2.01** | **7.47** | **0.70** | **.004** | **2.03** | **7.65** | **0.88** | **.021** |
| Age | **-.14** | **.87** | **0.06** | **.030** | 0.00 | 1.00 | 0.07 | .984 |  | -0.11 | 0.90 | 0.064 | .102 | 0.037 | 1.04 | 0.07 | .600 |
| Smoker at T1^b^ | **5.69** | **>100** | **0.36** | **<.001** | **7.08** | **>100** | **0.46** | **<.001** |  | **5.51** | **>100** | **0.45** | **<.001** | **6.62** | **>100** | **0.49** | **<.001** |
| Loneliness ^C^ | .14 | 1.15 | 0.19 | .482 | 0.17 | 1.19 | 0.23 | .455 |  | 0.02 | 1.02 | 0.20 | .935 | **0.48** | **1.62** | **0.23** | **.033** |
|  | al-RISCO T1-T2 (*N* = 404) | | | | | | | |  | al-RISCO T1-T2 (*N* = 404) | | | | | | | |
|  | Occasional smoker T2 | | | | Regular smoker T2 | | | |  | Occasional smoker T2 | | | | Regular smoker T2 | | | |
|  | *B* | OR | *SE* | *p* | *B* | OR | *SE* | *p* |  | *B* | OR | *SE* | *p* | *B* | OR | *SE* | *p* |
| Gender Male^a^ | 0.06 | 0.70 | 0.53 | .480 | 0.42 | 1.53 | 0.56 | .451 |  | -0.42 | 0.66 | 0.51 | .402 | 0.42 | 1.52 | 0.54 | .435 |
| Academic university^d^ | 0.35 | 1.42 | 0.73 | .634 | -0.64 | 0.53 | 0.65 | .320 |  | 0.66 | 1.93 | 0.72 | .360 | 0.56 | 1.75 | 0.66 | .400 |
| Age | -0.08 | 0.92 | 0.13 | .518 | 0.02 | 1.02 | 0.13 | .867 |  | 0.15 | 1.17 | 0.11 | .152 | -0.2 | 0.98 | 0.13 | .862 |
| Occasional smoker T1^e^ | **4.16** | **64.30** | **0.88** | **<.001** | **4.08** | **>100** | **0.65** | **<.001** |  | **3.15** | **23.40** | **.73** | **<.001** | **3.16** | **23.55** | **0.68** | **<.001** |
| Regular smoker T1^e^ | **2.38** | **10.84** | **0.73** | **.007** | **5.55** | **>100** | **0.65** | **<.001** |  | **2.76** | **15.84** | **0.73** | **<.001** | **5.28** | **>100** | **0.64** | **<.001** |
| Loneliness^f^ | -0.36 | 0.92 | 0.51 | .480 | 0.01 | 1.01 | 0.52 | .986 |  | -.55 | 0.58 | 0.49 | .257 | 0.91 | 2.48 | 0.13 | .073 |

*Note.* ^a^ The reference category for gender was female; ^b^ Due to model fitting problems the occasional and regular smokers were combined into 1 category of smokers. The reference category was non-smokers at T1. ^c^ Loneliness was measured with the Roberts UCLA Loneliness Scale (RULS-8) with mean scores ranging from 0 till 4. ^d^ Education level was coded as 1 for academic university (in Dutch “WO”), and 0 for applied university (in Dutch “HBO”) which was used as reference category. ^e^ The reference category was non-smokers at T1. The explained variance (Nagelkerke R-square) was 64.5% for HSL T1-T2, 56.8% for HSL T1-T3, 55.7% for al-RISCO W1-W2 and 46.9% for al-RISCO T1-T3. ^f^ Loneliness was measured with the Three-Item Loneliness Scale (UCLA-revised) with a mean score ranging from 1 till 3. HSL = Healthy Student Life project, al-RISCO = al-RISCO lifestyle project.

X. **Results sensitivity analyses**

*Adolescent samples (Samples 1, 2 and 3)*

Not applicable.

***Sample 4: Healthy Student Life project (HSL)***

The first sensitivity analysis on complete cases did not confirm the significant effects of baseline loneliness on smoking status at T3 as found in the main analysis (β = .10, *SE* = 0.09, *p* = .057), see Supplementary Table VI.1. Additionally, the first sensitivity analysis did not confirm significant effects of gender on smoking status at T2 (β = .06, *SE* = 0.22, *p* = .329). In line with the main analysis, we did find that smoking status at T1 did predict smoking status at T2 (β = .71, *SE* = 0.13, *p* < .001) and T3 (β = .63, *SE* = 0.11, *p* < .001).

The second sensitivity analysis where smoking status was replayed by smoking quantity was not in line with the main analysis as baseline loneliness did not predict smoking quantity status at T3 (β= .03, *SE* = 3.01, *p* = .716) as found for smoking status in the main analysis, see Supplementary Table VI.2. Additionally, the second sensitivity analysis did not confirm significant effects of gender on smoking quantity at T2 (β= .03, *SE* = 2.56, *p* = .357). In line with the main analysis, we did find that smoking status at T1 did predict smoking quantity at T2 (β = .82, *SE* = 0.09, *p* < .001) and T3 (β = .71, *SE* = 0.07, *p* < .001).

***Sample 5: al-RISCO lifestyle project (al-RISCO)***

The results of our sensitivity analysis were completely in line with the results from the main analysis confirming that smoking status at T1 did predict smoking status at T2 (β = .62, *SE* = 0.14, *p* < .001) and T3 (β = .57, *SE* = 0.15, *p* < .001), Supplementary Table VI.1. Similar to the main analysis, no additional effects were found in the sensitivity analysis.

**Supplementary Table IX.1.**

*Structural Equation Models for the Student Samples – Sensitivity Analysis 1 Complete Cases*

|  | HSL (*N* = 945) | | | | | | | |  | Al-RISCO (*N* = 321) | | | | | | | |
| --- | --- | --- | --- | --- | --- | --- | --- | --- | --- | --- | --- | --- | --- | --- | --- | --- | --- |
|  | Smoking status T2 | | | | Smoking status T3 | | | |  | Smoking status T2 | | | | Smoking status T3 | | | |
| T1 variables | *b* | β | *SE* | *p* | *b* | β | *SE* | *p* |  | *b* | β | *SE* | *p* | *b* | β | *SE* | *p* |
| Gender^a^ | 0.22 | .06 | 0.22 | .329 | -0.07 | -.02 | 0.18 | .708 |  | 0.36 | .13 | 0.29 | .220 | 0.21 | .08 | 0.25 | .394 |
| Education level^b^ | n.a. | n.a. | n.a. | n.a. | n.a. | n.a. | n.a. | n.a. |  | -0.09 | -.02 | 0.31 | .776 | 0.31 | .09 | 0.28 | .268 |
| Age | -0.01 | -.02 | 0.04 | .741 | -0.01 | -.02 | 0.03 | .740 |  | -0.04 | -.06 | 0.05 | .378 | -0.05 | -.08 | 0.05 | .355 |
| Smoking status^c^ | **2.26** | **.71** | **0.13** | **<.001** | **1.79** | **.63** | **0.11** | **<.001** |  | **1.55** | **.62** | **0.14** | **<.001** | **1.36** | **.57** | **0.15** | **<.001** |
| Loneliness | 0.08 | .04 | 0.10 | .453 | 0.17 | .10 | 0.09 | .057 |  | -0.02 | -.01 | 0.29 | .935 | 0.08 | .03 | 0.20 | .695 |

*Note.* ^a^Gender was coded as 0 for female and 1 for male; ^b^Education level was coded as 1 for applied university (in Dutch “HBO”) and 2 for academic university (in Dutch “WO”). ^c^Smoking status was coded as an ordered factor, with never smokers as 0, occasional smokers as 1, and frequent smokers as 2. n.a. = not applicable, HSL = Healthy Student Life project, al-RISCO = al-RISCO lifestyle project.

**Supplementary Table IX.2**

*Structural Equation Models – Sensitivity Analyses 2 Smoking quantity (in baseline smokers only; n = 247) in HSL Sample*

|  | Smoking quantity T2 | | | | Smoking quantity T3 | | | |
| --- | --- | --- | --- | --- | --- | --- | --- | --- |
| T1 variables | *b* | β | *SE* | *p* | *b* | β | *SE* | *p* |
| Gender^a^ | 2.36 | .03 | 2.56 | .357 | 1.61 | .02 | 3.54 | .650 |
| Age | 0.56 | .04 | 0.65 | .391 | **1.89** | **.15** | **0.90** | **.035** |
| Smoking quantity | **0.82** | **.83** | **0.09** | **<.001** | **0.68** | **.71** | **0.07** | **<.001** |
| Loneliness | -0.39 | -.01 | 2.11 | .853 | 1.10 | .03 | 3.01 | .716 |

*Note.* ^a^Gender was coded as 0 for female and 1 for male. HSL = Healthy Student Life project.

XI. **Results attrition analyses**

*Adolescent samples (Samples 1, 2 and 3)*

Not applicable.

*University Student samples (Samples 4 and 5)*

**Sample 4: Healthy Student Life project (HSL).** Logistic regression analyses showed that baseline age (*b* = -0.13, *SE* = 0.02, *p* < .001) and loneliness (*b* = -0.22, *SE* = 0.08, *p* = .004) predicted missingness on smoking status at T2, meaning that younger participants and participants who feel less lonely at baseline were more likely to miss data on smoking status at T2 (see Supplementary Table VII.1). Additionally, occasional smokers (vs. never and regular smokers) at baseline were more likely to miss data on smoking status at T2 (*b* = -0.52, *SE* = 0.22, *p* = .016) and both occasional (*b* = 0.58, *SE* = 0.21, *p* = .005) and regular smokers (*b* = 0.59, *SE* = 0.26, *p* = .022) at baseline were more likely to miss data on smoking status at T3.

**Sample 5: al-RISCO lifestyle project (al-RISCO).** Logistic regression analyses revealed that frequent smokers (vs. non-smokers) at baseline were more likely to miss data on smoking status at T3 (*b* = 1.19, *SE* = 0.41*, p* = .003) (see Supplementary Table VII.2). Additionally, baseline loneliness (*b* = -0.56, *SE* = 0.27, *p* = .039) predicted missingness on smoking status at T3, suggesting that participants who feel less lonely at baseline were more likely to miss data on smoking status at T3.

**Supplementary Table X.1**

*Logistic Regression for Attrition Analyses in HSL sample*

|  | Predicting missingness on smoking status T2 (*N*=2,170) | | | Predicting missingness on smoking status T3 (*N*=2,057) | | |
| --- | --- | --- | --- | --- | --- | --- |
| T1 variables | *b* | *SE* | *p* | *b* | *SE* | *p* |
| Loneliness | **-0.22** | **0.08** | **.004** | 0.03 | 0.06 | .623 |
| Smoking status |  |  |  |  |  |  |
| Occasional smoker vs. Never + Regular smoker | **0.52** | **0.22** | **.016** | **0.58** | **0.21** | **.005** |
| Regular smoker vs. Never + Occasional smoker | 0.16 | 0.30 | .605 | **0.59** | **0.26** | **.022** |
| Smoking quantity | <0.01 | 0.01 | .398 | -0.01 | 0.01 | .303 |
| Gender |  |  |  |  |  |  |
| Male vs. Female + Other | 0.09 | 0.13 | .490 | 0.20 | 0.11 | .069 |
| Other vs. Male + Female | -0.06 | 0.46 | .892 | 0.30 | 0.36 | .417 |
| Age | **-0.13** | **0.02** | **<.001** | 0.03 | 0.02 | .101 |

*Note*. Students who graduated between T1-2 and T1-3 were excluded from the attrition analyses. Smoking status at T1 was dummy-coded by creating two binary variables: Occasional smoker (1) vs. Never + Regular smoker (0); Regular smoker (1) vs. Never + Occasional smoker (0). HSL = Healthy Student Life project.

**Supplementary Table X.2**

*Logistic Regression for Attrition Analyses in al-RISCO sample*

|  | Predicting missingness on smoking status T2 (*N* = 402) | | | Predicting missingness on smoking status T3 (*N* = 402) | | |
| --- | --- | --- | --- | --- | --- | --- |
| T1 variables | *b* | *SE* | *p* | *b* | *SE* | *p* |
| Gender | 0.37 | 0.42 | .385 | -0.04 | 0.28 | .886 |
| Education level | -0.57 | 0.48 | .234 | 0.20 | 0.37 | .582 |
| Age | -0.18 | 0.12 | .131 | -0.13 | 0.08 | .099 |
| Smoking status |  |  |  |  |  |  |
| Occasional vs. Non-smoker + Frequent | 0.78 | 0.67 | .240 | -0.03 | 0.57 | .956 |
| Frequent vs. Non-smoker + Occasional | -0.05 | 0.78 | .945 | **1.19** | **0.41** | **.003** |
| Loneliness | -0.36 | 0.41 | .384 | **-0.56** | **0.27** | **.039** |

*Note.* Categorical variables were coded to facilitate logistic model interpretation. Gender at T1 was coded as 0 for women and 1 for men; Education level at T1 was coded as 1 for HBO and 2 for WO; Smoking status at T1 was dummy-coded by creating two binary variables: Occasional (1) vs. Non-smoker + Frequent (0); Frequent (1) vs. Non-smoker + Occasional (0). al-RISCO = al-RISCO lifestyle project.

*References:*

Harakeh, Z., Scholte, R. H., De Vries, H., & Engels, R. C. (2005). Parental rules and communication: Their association with adolescent smoking. *Addiction*, *100*(6), 862–870.

Rubio, M., Cillessen, A.H.N., Luijten, M., Vink, J.M. & Verhagen, M. (2024). From risk to resilience? Hazardous drinking trajectories in and beyond the last years of university life. *Emerging Adulthood*, *12* (5), 726-742. doi: [10.1177/21676968241273107](https://doi.org/10.1177/21676968241273107)

van den Broek, N., De La Haye, K., Veldhuis, L., Verhagen, M., Larsen, J. K., Vink, J. M., & Burk, W. J. (2022). Examining food intake similarities in adolescent best friend dyads using longitudinal Actor-Partner Interdependence Models. *Appetite*, *175*, 106072. https://doi.org/10.1016/j.appet.2022.106072

van den Broek, N., Larsen, J. K., Verhagen, M., Burk, W. J., & Vink, J. M. (2020). Is Adolescents’ Food Intake Associated with Exposure to the Food Intake of Their Mothers and Best Friends? *Nutrients*, *12*(3), 786. https://doi.org/10.3390/nu12030786

van Hooijdonk, K. J. M., Simons, S. S. H., van Noorden, T. H. J., Geurts, S. A. E., & Vink, J. M. (2023). Prevalence and clustering of health behaviours and the association with socio-demographics and mental well-being in Dutch university students. *Preventive Medicine Reports*, *35*, 102307. https://doi.org/10.1016/j.pmedr.2023.102307
